# We Call Our Colonel 'Charlie,' and We Obey Orders

**Published:** 1918-08-03

**Authors:** 


### HER MAJESTY THE MATRON.

### "We Call our Colonel 'Charlie,' and We Obey Orders."

There are always obstacles in the path of progress. This is a rule to which in this cosmogony of ours there is probably no exception, and on the whole the fact is not regrettable. To be good is troublesome, and if it were otherwise there would be no such quality as virtue. Nobody gives credit to the eagle for finding a home on the lofty mountain crag, but the mere biped who arrives there is a hero.

70,000 Dumb Women.

The British nurse, let us hope, has entered on the thorny path of progress, and she finds herself, at the start, inarticulate. And from an outside point of view an army of seventy thousand women, all dumb, is an extraordinary anomaly. There is nothing like unto it on all the earth. Quite a number of people talk on their behalf, and some there are who screech. But the patient, toiling woman who does the actual nursing passes across the stage in dumb show. She breakfasts at six or seven in the morning, puts out her candle at ten or thereabouts, having in the interval taken sufficient food and exercise to keep her body and soul in health, and for the rest of the day she is ministering to the needs of the sick. She is praised for her patience, preached at lest she should relax, but, owing to some peculiar and potent influence, is voiceless. The most silent and uncomplaining woman in the world is the British nurse. But she is repressed. She must be. Seventy thousand dumb women, naturally endowed with the gift of speech, is a phenomenon that cannot otherwise be explained.

An Overwhelming Sea of Silence.

I desire, if possible, to solve the problem. I confess that I approach it with a feeling of awe, for the overwhelming sea of silence gives one cause to shiver. It appears to me a thin-ice problem, so thin that I am obsessed of the danger. I almost fear the sound of my own voice. If, therefore, I should happen to flounder in my quest after truth I hope that somebody kindly and generously disposed will venture to throw me a rope, or else point me to some remote and hidden pathway by which I may retire into obscurity. For I have arrived at the conclusion that the main obstacle in the way to articulation is Her Majesty the Matron. I am in no respect dogmatic. I am, indeed, apologetic. I share with the candidate-probationer my inherent, immediate and ultimate fear of the absolute and despotic monarch of the hospital.

A Hard-worked and Ill-paid Woman.

Let it not for a moment be supposed that I propose to speak unkindly of the hospital matron. I know her too well. She is a hardworked and ill-paid woman, bearing on her shoulders a heavy load of responsibility. By night and by day she must think of the sick and suffering patients in her charge. For the veriest trifle that goes wrong the matron is responsible. The physician and the surgeon come along, and their cautions and directions are confided to her. If the charwoman should have a quarrel with the cook, they appeal not jointly but severally to her for arbitration and settlement. The hospital board assemble and discuss the affairs of the institution, and the most trivial incident of waste or carelessness or discord is automatically referred to the matron. The members are chiefly gentlemen of leisure, and sometimes ladies of leisure, and the hospital management is a mere incident of their lives. I have peeped in at some of these meetings, and the prevailing notes at the best of them are two—efficiency and finance. So interlaced are these that it is impossible to give priority to either. A hospital chairman's keynote is "I have to find the money." He cannot find the money if the hospital is inefficient. He wants all that he can gather in, and so he hires out his nurses. The nurses are the backbone of the whole establishment, and so he, and some other boards like his, form a *liaison* with the nursing staff through the medium of the matron, herself a nurse. And so it works out that in a community of nurses the hospital matron is in actual practice a working nurse, badly paid, loaded with responsibility and care, armed with a whip.

What is the Matron's own View?

Examine and perceive the thin ice on which I dare to tread. Here is a woman whose personal character must of necessity be white as the driven snow, whose ability in nursing, in economy, in discipline, and in general management must be of the highest, and who must, at the peril of her appointment, keep her staff in absolute control. To describe her, therefore, as an obstacle in the path of progress is obviously to place her in a very invidious and undesirable position. Yet this is exactly where I am, and I am sorry to say it. I have a feeling amounting to conviction that the matron is responsible for the silence of the nurse. Her business in life is to maintain the efficiency of the hospital, and at the same time to keep the staff dumb—to constrain, perhaps in a very nice way, into willingness to work through long hours and weary days without complaint, and all in the interest of the nation's public and private philanthropy. I am unable to estimate to what extent matrons themselves, as well as the rank and file, will agree with my contention. Perhaps some of them may be so good as to tell me. Even a violent diatribe would be a pleasant break in the monotony of stony silence.

Really a Question of Discipline.

The question is really one of discipline, and there is no more severe and exacting a disciplinarian than a woman reared in a hard school. These matrons have been through the mill. Many of them wept themselves to sleep when they were probationers, and now when they are enthroned they imitate the tyrants at whose passing glance they used to quake. They carry the scourge that once on a time they looked upon with terror in their hearts. I am not a Bolshevik, I have no sympathy with soldiers holding committee meetings on the eve of battle. But neither am I in favour of chaining artillerymen to the guns lest they should desert. Let me illustrate. I happened to be sipping tea recently with two Canadian Tommies, and we got on the subject of discipline. They were fine strapping young soldiers, full of war enthusiasm, but they laughed to scorn our Army's disciplinary code, and this is how one of them summed up the situation: "Why, you know," he said, "we call our Colonel 'Charlie,' but he is our Colonel, and we obey orders." This may seem an extraordinary state of affairs, but it reveals the possibility of comradeship and discipline going hand in hand to battle.

How often, or how seldom, may I inquire, is the matron the comrade of the nurse? In my personal experience, occasionally—not often, and very seldom. Her Majesty the Matron, as a rule, is, in the young nurse's estimate, the woman with the whip!

